# Topological Phenotypes Constitute a New Dimension in the Phenotypic Space of Leaf Venation Networks

**DOI:** 10.1371/journal.pcbi.1004680

**Published:** 2015-12-23

**Authors:** Henrik Ronellenfitsch, Jana Lasser, Douglas C. Daly, Eleni Katifori

**Affiliations:** 1 Max Planck Institute for Dynamics and Self-Organization, Göttingen, Germany; 2 New York Botanical Garden, Bronx, New York, United States of America; 3 Department of Physics and Astronomy, University of Pennsylvania, Philadelphia, Pennsylvania, United States of America; University of Alberta, CANADA

## Abstract

The leaves of angiosperms contain highly complex venation networks consisting of recursively nested, hierarchically organized loops. We describe a new phenotypic trait of reticulate vascular networks based on the topology of the nested loops. This phenotypic trait encodes information orthogonal to widely used geometric phenotypic traits, and thus constitutes a new dimension in the leaf venation phenotypic space. We apply our metric to a database of 186 leaves and leaflets representing 137 species, predominantly from the Burseraceae family, revealing diverse topological network traits even within this single family. We show that topological information significantly improves identification of leaves from fragments by calculating a “leaf venation fingerprint” from topology and geometry. Further, we present a phenomenological model suggesting that the topological traits can be explained by noise effects unique to specimen during development of each leaf which leave their imprint on the final network. This work opens the path to new quantitative identification techniques for leaves which go beyond simple geometric traits such as vein density and is directly applicable to other planar or sub-planar networks such as blood vessels in the brain.

## Author Summary

Planar reticular networks are ubiquitous in nature and engineering, formed for instance by the arterial vasculature in the mammalian neocortex, urban street grids or the vascular network of plant leaves. We use a topological metric to characterize the way loops are nested in such networks and analyze a large database of 186 leaves and leaflets, revealing for the first time that the nesting of the networks’ cycles constitutes a distinct phenotypic trait orthogonal to previously used geometric features. Furthermore, we demonstrate that the information contained in the leaf topology can significantly improve specimen identification from fragments, and provide an empirical growth model that can explain much of the observed data. Our work can improve understanding of the functional significance of the various leaf vein architectures and their correlation with the environment. It can pave the way for similar analyses in diverse areas of research involving reticulate networks.

## Introduction

The angiosperm leaf vein network fulfills the combined requirements of efficient liquid transport within the leaf and high robustness against load fluctuations and damage, while at the same time providing structural reinforcement [1–4].

Modern leaf vein networks evolved gradually from simple dendritic branching patterns by introduction of anastomoses [5, 6], leading to leaf vascular networks that are highly reticulate, exhibiting nested, hierarchically organized vein loops. The reticulate leaf vascular system is an example of evolutionary adaptation under various constraints [1, 7–10].

**Fig 1:**
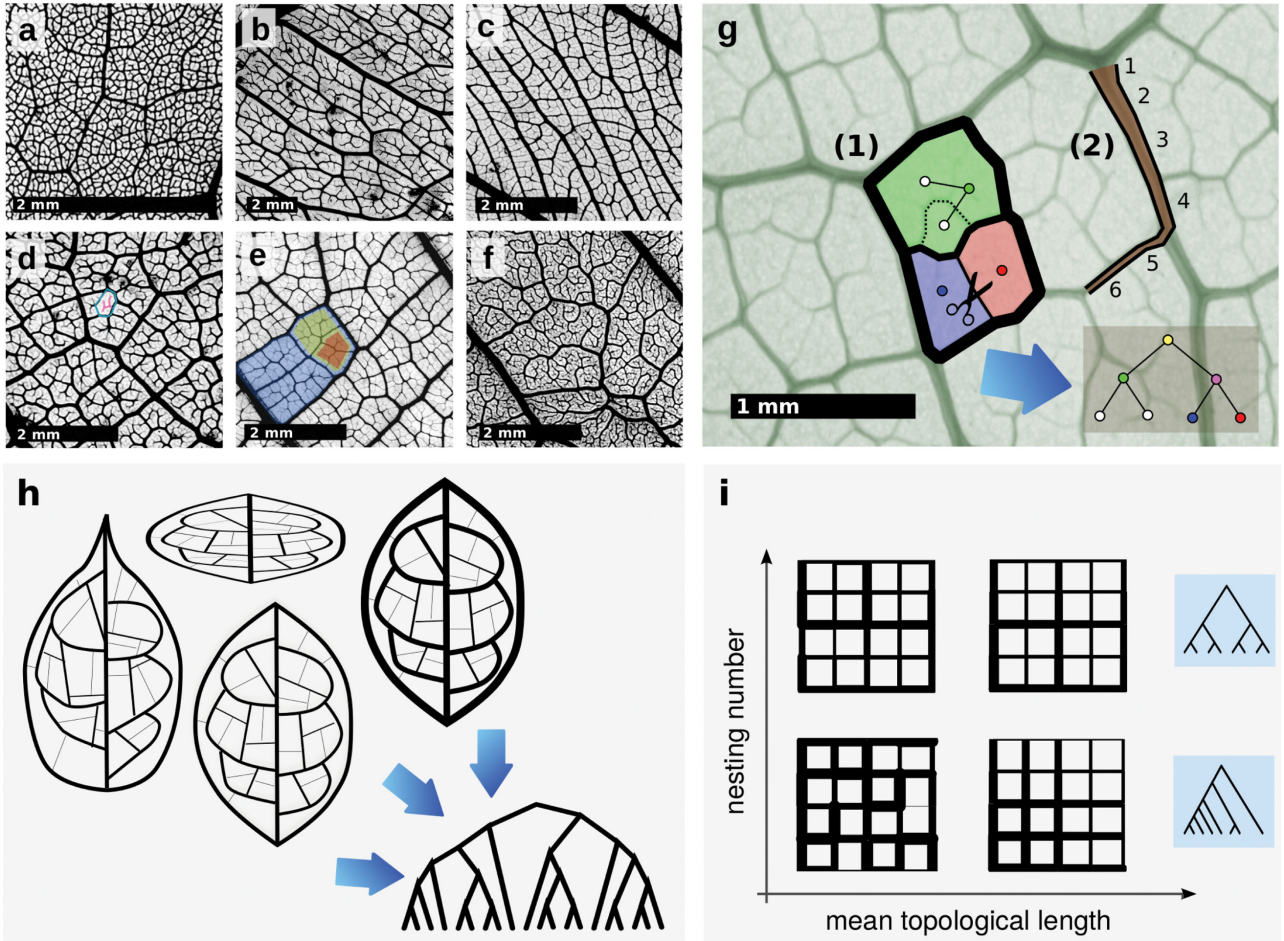
**a-f** Diversity of high order leaf venation within the Burseraceae. **a** *Protium ovatum*. **b** *Protium madagascariense*. **c** *Pouteria filipes*. **d** *Canarium betamponae*. A single areole is marked in blue, non-anastomosing highest order veins in red. **e** *Brosimum guianensis*. The hierarchical nesting of loops is highlighted. **f** *Protium subserratum*. **g** (1) The hierarchical decomposition algorithm. In a pruning step, all subgraphs which cannot be expressed as a superposition of cycles are removed. Then, the areoles are identified (facets) and assigned to leaf nodes in a tree graph (circles). The two facets whose intersection has minimum weight (vein thickness) are detected, their intersection is removed and their leaf nodes are connected to a new node which is identified with the new loop. The procedure is iterated until only one facet is left, which is identified with the root node. (2) Topological tapering length. Starting from a thick edge, we walk on the network to that adjacent edge with the largest diameter that is still smaller than that of the current edge. This is repeated until no edge to proceed to can be found, and the number of edges traversed is counted. **h** Illustration of different leaf networks which result in the same hierarchical decomposition tree. Networks may undergo a geometric transformation such as stretching or squeezing. The vein thicknesses may change as long as their relative rank remains the same. Positions of junctions may change provided that no other junctions are crossed. **i** The space of topologies described by mean topological length and nesting number. Shown are typical networks exhibiting various combinations of vein tapering and loop nestedness, as well as typical associated nesting trees.

Despite some common trends, the diversity of vein morphology in dicotyledonous plants is striking (see for instance Fig 1a–1f). Current models of vascular development in the model species *Arabidopsis thaliana* predict several overlapping phases in which the leaf primordium at first mainly grows by cell division, then later by cell expansion [3, 11]. Lower order (major) veins are thought to be formed during the first phases, whereas minor veins are formed primarily during the later, leaving an imprint in the higher order vascular system of the leaf.

The morphology, anatomy, and correlations with climate of the lower order vascular architecture have been extensively studied [12, 13], and primary and secondary vein traits can be easily quantified [3]. Certain leaf traits such as vein density are closely linked to photosynthetic efficiency [14–16]. Links to climatic conditions and vegetation type have been proposed as well [5, 13, 17, 18].

The hydraulic resistance of the whole plant is strongly affected by the leaf hydraulic resistance. The smallest veins, by virtue of their combined length and small hydraulic diameter are responsible for the bulk of this resistance. At the same time, the smallest veins, and in particular the small free-ending veinlets, are perhaps the most crucial for water delivery [19]. However, the architecture of higher order vein reticulation has been largely ignored in the literature. Other than an extensive descriptive nomenclature [20] and mainly qualitative measures [21], to this day there is no quantitative work that goes beyond obvious geometric characteristics, like minor vein density, areole size, angle distribution, vascular segment length and width distribution [3, 22, 23]. These characteristics by themselves are not sufficient to describe the full architecture, in particular the organization of the loops. Loops typically show a large degree of hierarchical nesting, i.e. larger loops composed of larger-diameter veins contain many smaller loops with smaller vein diameter (see Fig 1e).

Although topological studies of spatial network architectures such as street networks are quite common [24], a detailed quantitative characterization of the topological properties related to reticulation has been elusive in the past, and only recently have researchers started to seriously attack the question [23, 25, 26].

We use ideas inspired by computational topology [27] to define a metric suitable to quantify the architecture of higher order venation of leaves. We apply our topological metric to a dataset of 186 leaves and leaflets, demonstrating that our characterization constitutes a new phenotypic trait in plant leaves and carries information complementary to previously used quantities. We then show that this information can be useful in the task of identifying leaves from fragments, significantly improving identification accuracy. We finally present a growth model that reproduces most of the observed variation in the topological traits.

Our results suggest that topological and geometric venation traits are approximately independent, and that the higher order venation topology is mainly controlled by a small set of parameters regulating noise during vein morphogenesis.

The topological venation traits we use can be employed in much broader contexts than leaves, being applicable to any (sub-)planar, anastomosing network such as blood vessels in the brain, liver or retina, foraging networks build by slime molds, lowland river networks, urban street networks or force chains in granular media, thereby possibly opening up an entire new line of research.

### Topological phenotypes

Our topological metric quantifies the hierarchical nesting of loops within the network as well as the topological lengths of tapered veins. The analysis follows an existing hierarchical decomposition algorithm [25, 26, 28], constructing from a weighted network a binary tree graph termed the *nesting tree* which contains information about nesting of loops. The algorithm is schematically shown in Fig 1g and discussed in the supplement.

We stress that the method depends not on exact measurements of vein diameters but only on relative order. Similarly, transformations which slightly alter node positions do not affect the outcome (see Fig 1h).

Once the binary nesting tree (see Fig 1g) has been obtained, its structure can be quantified. Here, for each node *j* in the nesting tree, we calculate the nesting ratio $q_{j}=\frac{s_{j}}{r_{j}}$[29], where *r*_*j*_ ≥ *s*_*j*_ are the numbers of leaf nodes in the right and left subtrees of node *j*. We then define the *nesting number* as a weighted average *i* = ∑_*j*_ *w*_*j*_ *q*_*j*_, where ∑_*j*_ *w*_*j*_ = 1. We employ an unweighted nesting number *i*_*u*_, with *w*_*j*_ = 1, and a degree-weighted nesting number *i*_*w*_, with *w*_*j*_ ∝ *d*_*j*_ − 1 = *r*_*j*_ + *s*_*j*_ − 1, where *d*_*j*_ is called *subtree degree*. A high value of *i*_*u*,*w*_ qualitatively represents graphs that are highly nested such as those in the top row of Fig 1i.

The presence and extent of tapered veins is quantified as follows. Starting from some edge *e*, we find the next edge by taking the maximum width edge amongst all with smaller width than *e*. We count how many steps can be taken until no more edges with smaller width are adjacent, resulting in a topological length *L*_*e*_ assigned to each edge in the network. The mean topological length $L_{\text{top}}=\frac{1}{N_{E}}\sum_{e}L_{e}$, where *N*_*E*_ is the number of edges, characterizes tapered veins in a network. Fig 1i shows a qualitative representation of various example network topologies using mean topological length and nesting number.

Instead of using just the nesting number, we additionally calculate pairwise topological distances between networks as the two-sample Kolmogorov-Smirnov statistic *D*_*KS*_ between the cumulative distributions of nesting ratios in order to quantify the statistical similarity between nested loop topologies. Other methods to quantify the degree of topological dissimilarity between binary trees representing biological systems have been proposed on the basis of a “tree edit distance” [30]. Despite promise, this distance suffers from being dominated by differences in the size of the compared trees. In its local form [31], it suffers from the opposite problem, quantifying only the similarity between the *n* most similar subtrees. In contrast, our method is designed to capture statistical similarities between nesting trees, making it more suitable for dissimilarly sized, noisy networks.

## Results

We show that the topological characteristics described above provide a new dimension in the phenotypic space of leaf venation morphology.

For this, we analyze a dataset consisting of 186 leaflets from various species primarily belonging to the Burseraceae family (see S1 Text and S1 Table). Although most of species are therefore closely related, their venation patterns show considerable diversity (see Fig 1a–1f), rendering them a good test set for our metrics The leaves were chemically cleared and stained to make their higher order venation network apparent [32], then scanned at high resolution (6400 dpi) and vectorized in-house (see S1 Text). Scanning whole leaves and digitizing at high resolution is computationally expensive but necessary for this work to accurately represent the statistics of the high order veins [33]. Publicly available databases of scanned specimens [34] contain mostly low resolution images.

### Analysis of full leaf networks

**Fig 2:**
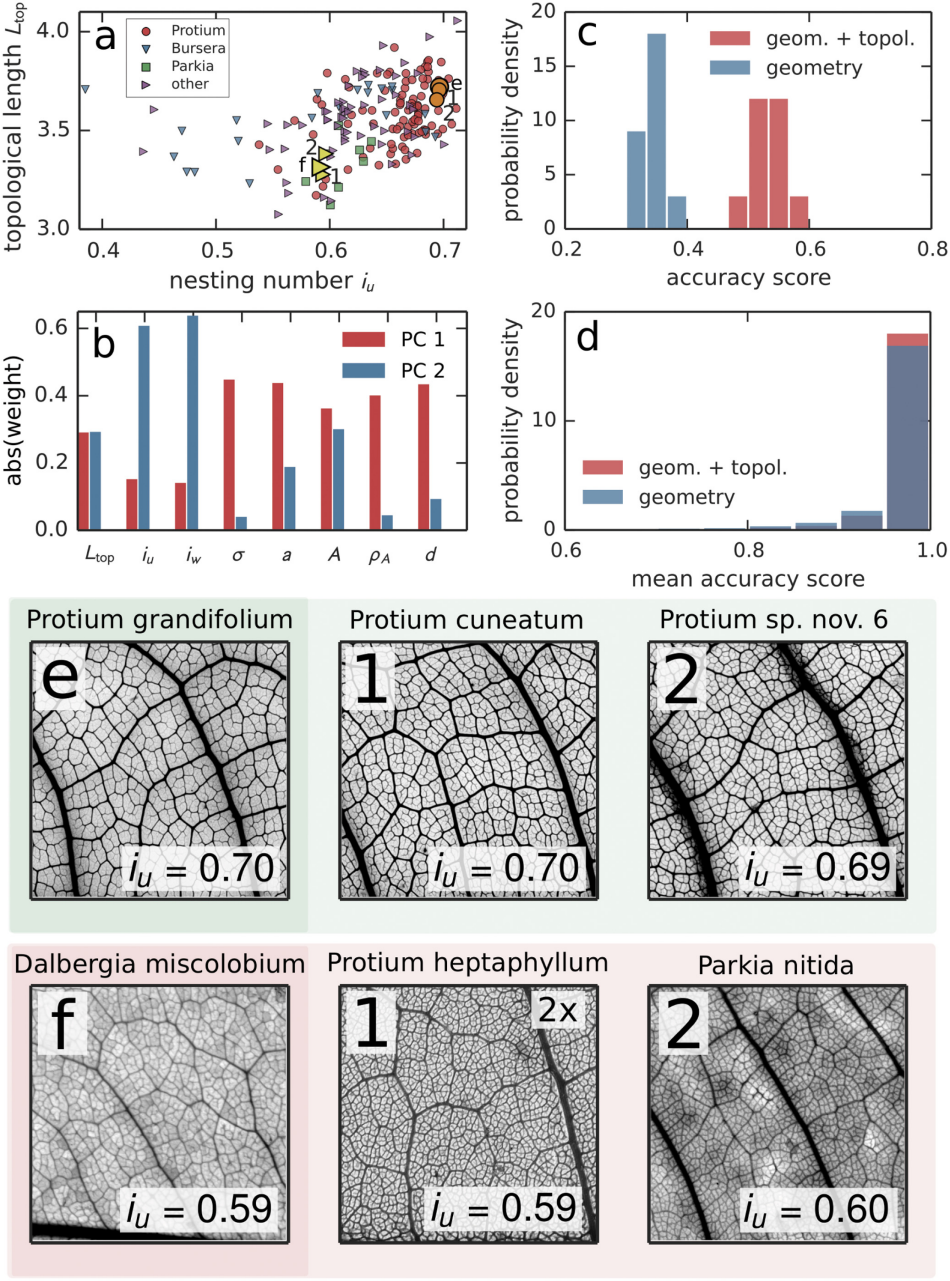
**a** Plot of the whole dataset consisting of 186 leaf networks depending on the unweighted nesting number *i*_*u*_ and mean topological length. One leaf of *Protium grandifolium* (*Dalbergia miscolobium*) is marked by a circle (triangle) with black border as well as shown in e (f). The smaller circles (triangles) in the same color show the two nearest neighbors according to the statistical distance *D*_KS_. The specimen belonging to the most abundant genera in the dataset are marked in order to assess predictive accuracy at a higher taxonomic level. The specimen belong to *Protium* (98 specimen), *Bursera* (21 specimen) and *Parkia* (8 specimen). **b** Weights of the 8 metrics, in the first two principal components of the dataset. Component 1 contains mostly geometry (*σ*, *a*, *A*, *ρ*_*A*_, *d*), Component 2 mostly topology (*L*_top_, *i*_*u*_, *i*_*w*_), see also S1 Text. **c** Results of leaf identification from fragments using Linear Discriminant Analysis (LDA). Accuracy scores were obtained using 10-fold stratified cross-validation. The plot shows histograms of the resulting accuracy scores. Accuracy of identification is significantly improved when using both geometrical and topological information as opposed to only geometry. (Welch’s *t*(15.6) = 15.8, *p* < 0.001). **d** Summary results of pairwise leaf identification from fragments. All pairs of leaves were classified individually using LDA. Again, using topological traits significantly improves the summary result (see S1 Text). **e, f** Images of the same leaves as those specially marked in **A** and their nesting numbers *i*_*u*_, together with their nearest two neighbors 1, 2. All images except for **f-1** show a 1cm × 1cm gray-scaled and contrast-enhanced crop of the original scan. Image **f-1** was zoomed in by a factor of 2 to show the nesting structure more clearly.

From the vectorized data, we obtained for each leaf five local geometric quantities: vein density *σ* (total length of all veins/leaf area), mean distance between veins *a*, mean areole area *A*, areole density *ρ*_*A*_, and average vein diameter weighted by length of venation between junctions *d*. The (un)weighted nesting number *i*_(*u*)*w*_ was calculated from all subtrees of the nesting tree with degree *d* ≤ 256 in order to remove leaf size effects for the full networks; the mean topological length was calculated from the whole network. Together, these metrics form a “leaf venation fingerprint” encompassing *local* features of the network, that can be estimated from leaf segments alone if necessary. Fig 1a shows the complete dataset plotted in the space of unweighted nesting number and mean topological length. We plot the most abundant genera *Protium* (98 specimen in the dataset), *Bursera* (21 specimen), and *Parkia* (8 speciment) as different symbols. Although the dataset does not allow for firm conclusions at this taxonomic level, both *Protium* and *Parkia* appear to show a modest trend towards clustering around characteristic nesting numbers. We then employed Principal Component Analysis (see Fig 2b) and found that together, the first two principal components explain 81% (= 52% + 29%) of the total variance in the dataset. Component 1 can be interpreted as containing mostly metrics derived from geometry, whereas Component 2 contains mostly metrics from topology. Topological lengths contribute roughly equally to either. Even though small correlations between them exist, this reveals local geometrical and topological leaf traits as approximately orthogonal traits for the description of the phenotype of leaf venation (see S1 Text, also for further analysis of the data in terms of latent factors).

Pairs of leaves (see Fig 2a and Fig 2e and 2f) which are close according to the topological distance defined by the *D*_*KS*_ metric applied to the nesting ratio statistics can possess similar “by eye” venation traits. In the samples in Fig 2e and 2f, cycle nestedness and vein thickness are traits that appear correlated. However, the topology of leaf venation constitutes a new phenotypic trait that provides information orthogonal to geometric traits.

### Analysis of leaf fragments

Topological information significantly helps in identifying leaf samples to species, especially when only a segment of the leaf is available. We fragmented all leaf samples in silico into equally sized segments of ca. 1.2 × 1.2cm and calculated all venation traits for the individual pieces (see S2 Table). Here, we thresholded the nesting ratios at subtree degree *d* ≤ 128. We employed Linear Discriminant Analysis (LDA) [35] to classify the fragments based on specimen membership (see also S1 Text). We then calculated the the probability of correctly identifying a segment as belonging to one of the 186 leaves and leaflets (the accuracy, see Fig 2c). Using only geometrical degrees of freedom, we found a 10-fold cross-validated accuracy of 0.35 (95% CI: [0.31, 0.39]). Adding topology improves the accuracy to 0.54 (95% CI: [0.48, 0.60]). Additionally, for each pair of individual leaves in the dataset, the same procedure was applied to obtain a mean pairwise accuracy score (the probability of correctly identifying a fragment as belonging to one of two leaves.) Again, using topological traits significantly improved the summary result (see Fig 2d and S1 Text). The same classification was applied towards identification of segments to species, as opposed to samples, with quantitatively similar results (see S1 Text).

It must be noted that there can be considerable variance among leaf traits, even when comparing among specimen from a single plant—in particular between sun- and shade leaves [6, 36]— that should be taken into account if the information is available.

### Comparison with venation growth model

In order to explain the nesting ratio and topological length distributions measured in our dataset, we examine a developmental model for the formation of higher-order venation in which the interplay between strictly hierarchical loop genesis and random noise is the major factor affecting nestedness.

**Fig 3:**
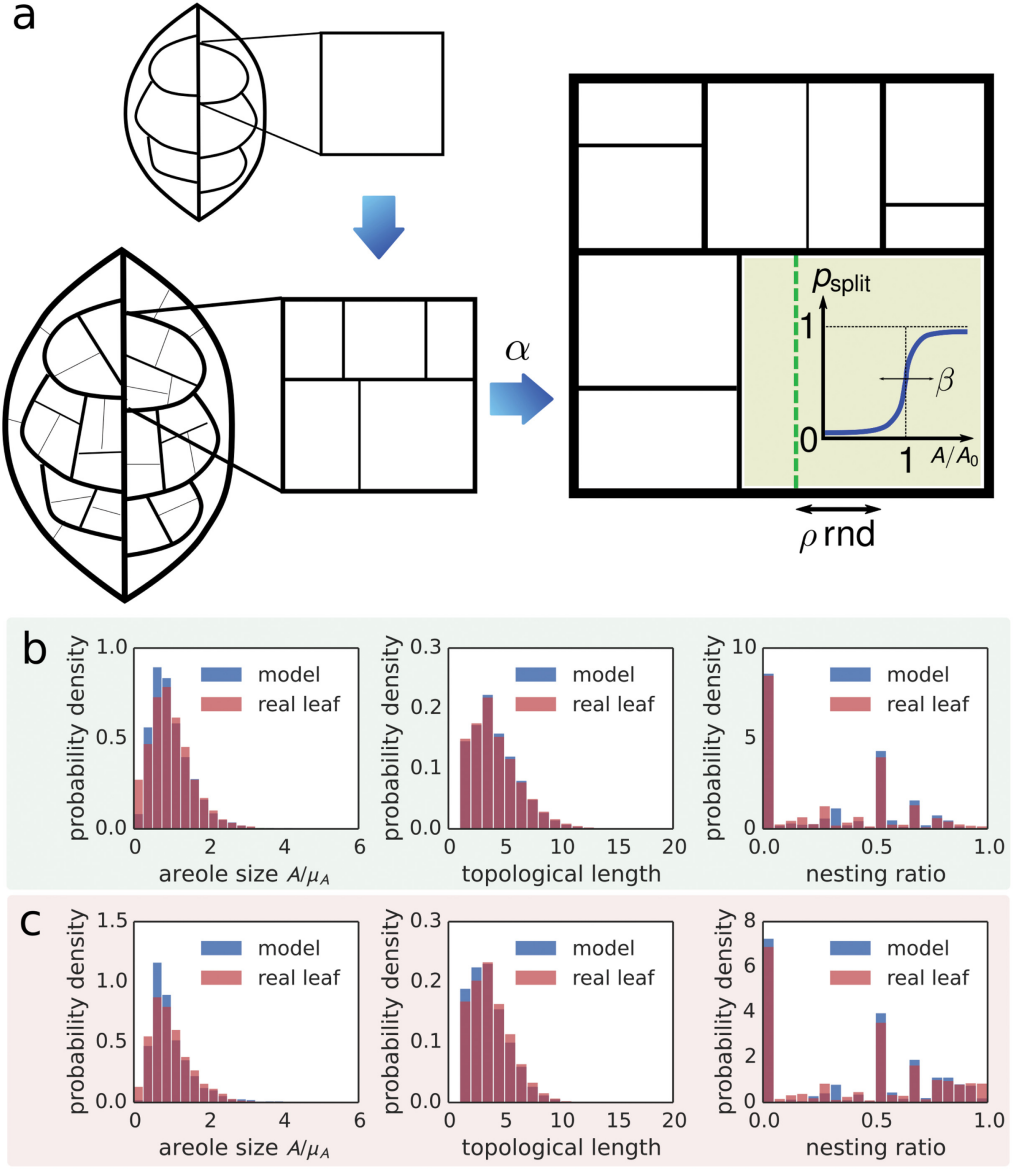
**a** Developmental growth model. Beginning from a rectangular loop, new veins are introduced by successively splitting existing loops. A new vein is formed at each iteration step with probability *p*_split_, which becomes significant as soon as loop area *A* is close to a critical loop area *A*_0_. *ρ*rnd determines the position of the new vein. **b** Comparison between developmental model and the same leaf as in Fig 2e, *Protium grandifolium*. We compare the distributions of areole size *A* normalized by the mean *μ*_*A*_, topological lengths and nesting ratios. The model parameters were *α* = 0.25, *β* = 0.5, *ρ* = 0.2, *f*_*n*_ = 0.1, a low noise setting. The distributions agree well, explaining the strongly hierarchically nested structure in the high level venation network of *P. grandifolium*. **c** Comparison between developmental model and the same leaf as in Fig 2f, *Dalbergia miscolobium*. The model parameters were *α* = 0.25, *β* = 0.3, *ρ* = 0.2, *f*_*n*_ = 0.45, a high noise setting. Again, the distributions agree reasonably well, explaining the relatively weakly nested high level venation network in *D. miscolobium* by a large amount of noise in the vein widths.

Empirically, during the expansion growth phase of the leaf lamina, high order vein loops grow and are subdivided by the appearance of new veins, subsequent vein orders appearing discretely one after the other [11, 37]. Our model intends to capture this phenomenological fact (see Fig 3a for an illustration). The model is compatible with models of vein morphogenesis that invoke either auxin canalization [38] or mechanical instabilities [39], or a combination. It is similar in spirit to that described in the supporting information of [39] or [40] but adds fine-grained control of stochasticity.

We stipulate that each leaf is subject to a species dependent characteristic amount of noise during development, resulting in unique characteristic statistics of minor venation patterns.

The model as a whole is controlled by four dimensionless parameters (see Methods section). In Fig 3b and 3c we show the distributions of normalized areole size, mean topological lengths and nesting ratios for the same two leaves as in Fig 2e and 2f. The real distributions can be explained well by tuning two of the parameters. Thus, noise during growth of cycles can explain the observed local hierarchical nesting characteristics.

It should be noted that different mechanisms may underlie the organization of low order veins. Indeed, both models [41] and empirical observations [42] have found strong links between low order vein structure and leaf shape that may be connected to the overall growth pattern and developmental constraints of the lamina [43].

## Discussion

The leaf vasculature is a complex reticulate network, and properly chosen and defined topological metrics can quantify and highlight aspects of the architecture that have been ignored until now. The topological metrics presented in this work provide a new, independent dimension in the phenotypic space of leaf venation, allowing for more precise characterization of leaf features and improved identification accuracy, including identification of fragments. The extensive nomenclature for characterization of the vascular morphology [20] offers a discrete set of attributes that is mathematically insufficient to properly quantify a continuum of leaf venation phenotypes. However, this descriptive terminology can be incorporated as additional topological dimensions in the phenotypic space and alongside the metrics presented in this work can provide a tool to quantify inter- and intra- species diversity. In addition, we show that the local hierarchy of nested loops in the leaf venation network can be explained by very simple stochastic processes during development, pointing toward a universal mechanism governing (minor) vein morphogenesis.

The topological measures we employ have possible applications that range far beyond the leaf data set explored here, being usable on any loopy complex weighted network which possesses an embedding on a surface. Examples of systems that could benefit from an analysis along the lines of this work include the blood vessels in the retina, liver or brain, anastomosing foraging networks built by slime molds and fungi, lowland river networks, human-made street networks, force chain networks in granular materials, and many more, thereby possibly opening up an entire new line of research.

## Materials and Methods

### Vectorization

The extraction the networks from the original high-resolution scans (6400 dpi) can be divided into two main steps: segmentation of the image to create a suitable binary representation and skeletonization of the shapes. To segment the image we use a combination of Gaussian blurring to reduce noise, local histogram equalization and recombination with the original image to increase contrast, and Otsu thresholding [44] to find the optimal threshold for the creation of the binary image. For the skeletonization we use a vectorization technique known from optical sign recognition [45, 46]. The approach relies on the extraction and approximation of the foreground feature’s contours using the Teh-Chin dominant point detection algorithm [47] and subsequent triangulation of the contours via constrained Delaunay triangulation [48]. Therefore the foreground is partitioned into triangles which can be used to create a skeleton of the shape. Each triangle contributes a “center” point to the skeleton which is determined by looking for local maxima in the euclidean distance map [49] of the binary and together these center points approximate the skeleton. By looking at edges shared between two triangles, neighborhood relations can be established and an adjacency matrix can be created. This adjacency matrix defines a graph composed of nodes (the former triangle centers) and edges (the connections between two adjacent triangles). In addition to the topology of the graph the original geometry of the network including coordinates of the nodes and lengths and radii of edges are preserved and stored in the graph. The processing is done using algorithms implemented in python. The framework uniting all the aforementioned functionality is freely available at [50].

### Hierarchical decomposition

A complete and detailed description of the hierarchical decomposition algorithm to extract the nesting tree from leaf network graphs can be found in the supplement S1 Text. The software package used to calculate nesting numbers, topological lengths, and geometric metrics is freely available at [51].

### Modeling cycle nesting

The model starts from a single rectangular loop of veins (Fig 3a). The loops grow and subdivide when they reach a threshold size *A*_0_ by introduction of a new vein. Not all loops subdivide at exactly the same size: the probability of subdivision as a function of areole area is a sigmoidal of width *σ*_*A*_ (Fig 3). All veins start with a fixed small width and grow linearly with time. The relative growth rate of vein lengths and widths is controlled by the nondimensional parameter *α*. The areole subdivision is only approximately symmetric: the new vein is randomly positioned close to the midline of the areole and the extent of the asymmetry is controlled by a parameter *ρ* ∈ [0, 1] (see S1 Text). After the growing leaf has a certain size, the simulation is terminated and random Gaussian noise with zero mean and standard deviation proportional to the parameter *f*_*n*_ is added to the vein diameters.

The model is controlled by the four dimensionless parameters *ρ*, *β* = *σ*_*A*_/*A*_0_, *α* and *f*_*n*_.

## Supporting Information

S1 Text Detailed description of methods and further analysis. Includes description of the geometric and topological metrics used including more explicit hierarchical decomposition algorithm, an explanation of the leaf clearing, staining and vectorization process, more details on the cycle growth model. Further data analysis includes comparison of our data set with earlier work, validation of the method, and detailed results of PCA and Factor Analysis.
(PDF)

S1 Table Leaf fingerprint database. The complete fingerprint data extracted from the full leaf networks.
(ODS)

S2 Table Leaf fragment fingerprint database. The complete fingerprint data extracted from the 1.2 × 1.2 cm leaf fragments.
(ODS)
