# Supplementary material for: Topological Phenotypes Constitute a New Dimension in the Phenotypic Space of Leaf Venation Networks
Source: PLoS Comput Biol. 2015 Dec 23;11(12):e1004680. doi: 10.1371/journal.pcbi.1004680 (PMC4699199; doi:10.1371/journal.pcbi.1004680)
Supplement: S1 Text — Includes description of the geometric and topological metrics used including more explicit hierarchical decomposition algorithm, an explanation of the leaf clearing, staining and vectorization process, more details on the cycle growth model. Further data analysis includes comparison of our data set with earlier work, validation of the method, and detailed results of PCA and Factor Analysis. (PDF) [file pcbi.1004680.s001.pdf]

Supplemental Material for "Topological Phenotypes of Leaf Venation Networks"

Henrik Ronellenfitsch, Jana Lasser, Douglas C. Daly, Eleni Katifori

November 27, 2015

1 The Leaf Dataset

The leaf dataset analyzed in the main paper consists of 207 vouchered leaves and leaflets collected by D. Daly, cleared, stained and mounted in resin at the New York Botanical Garden [1], most of them belonging to the frankinscence and myrrh family *Burseraceae*. After vectorization (see section 9), we extracted a number of local geometrical quantities for each leaf, as described in the following sections. We discard from the analysis all leaves with fewer than 256 areoles (either very small or badly stained/damaged specimens), leaving us with 186 good quality leaves to analyze.

2 Geometrical metrics

2.1 Vein density

Vein density σ is calculated by summing the total length of all veins and dividing by total leaf area. It is possible to also calculate the density of the main vein σ_{main}, or the secondaries σ_{sec} by summing up only the lengths of the respective veins, similar to [2].

Since manual measurement of vein lengths is cumbersome, we wrote an automatic program of detecting the main vein from vectorized images. The algorithm is as follows. We first calculate a weighted minimum spanning tree of the vein network with edge weights $w = 1/d$, where d is vein thickness. The main vein is then found as follows. (1) Identify the thickest edge e on the minimum spanning tree. (2) Identify the two thickest adjacent edges f_1, f_2. (3) Starting from f_1, f_2, successively find the thickest adjacent edges until no more edges are left. This set of edges is then defined as the main vein.

Detecting secondary veins is more cumbersome even though algorithmic methods exist [3]. Unlike the authors of [2] we forego this endeavor.

In this work we define *major* veins as all veins which consist of the $10 \times \sqrt{N}$ thickest veins, where N is the total number of veins (straight connections between two junctions) in the network. This definition only roughly captures the right proportion of veins, as this can vary between leaves with different numbers of veins. In Figure 1 we show the major veins according to this definition in a few leaf networks. Indeed, they overall correspond to the main vein, the secondary veins and sometimes some third order veins. The *minor* veins on the other hand we define by the lower 95% percentile of all vein thicknesses. This allows for calculation of σ_{major} which behaves similarly as the secondary vein density calculated in [2].

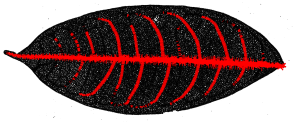

(a) *Protium grandifolium*

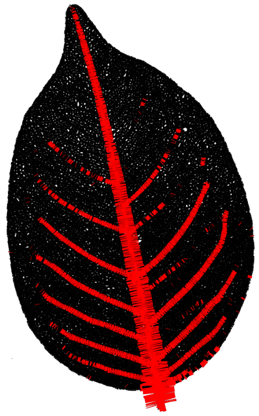

(b) *Apuleia leiocarpa*

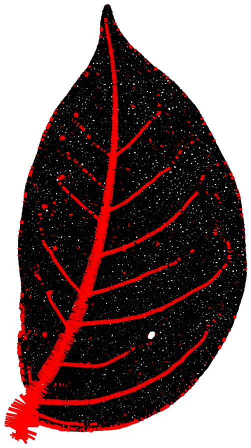

(c) *Hymenaea parvifolia*

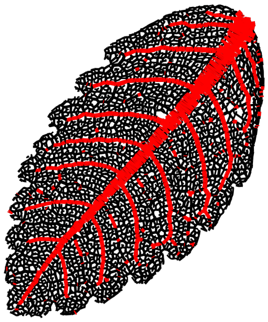

(d) *Bursera glabrifolia*

Figure 1: The major veins as defined by the $10 \times \sqrt{N}$ thickest veins in the network. We show 4 leaf networks from our data set. The major veins roughly correspond to the main vein, the secondary veins, and sometimes third order veins. The jagged lines are plotting artifacts.

2.2 Vein distance

The mean distance between veins a is estimated as follows. (a) Find all fundamental cycles (areoles) in the network. (b) For each areole, calculate the convex hull. (c) Find the Chebyshev center of the convex hull using a convex optimization procedure (we used the `cvxopt 1.1.7` package).[1] This effectively calculates the largest inscribed circle, whose diameter is taken as an estimate for vein distance.

2.3 Areole area

Mean areole area A is calculated by taking the fundamental cycles found for the vein distance calculation and calculating their areas using the standard formula for the area of a general polygon. It is

$$A = \frac{1}{2} \left| \sum_{i=0}^{k-1} x_i y_{i+1} - x_{i+1} y_i \right| . \tag{1}$$

Here, the sum runs over all k vertices in the closed polygon.

2.4 Areole density

Areole density ρ_A is simply the total number of areoles divided by leaf area.

2.5 Weighted vein thickness

Weighted vein thickness d is calculated as the average

$$d = \frac{1}{w} \sum_j d_j \, \ell_j, \quad w = \sum_j \ell_j, \tag{2}$$

where d_j are the thicknesses of individual veins (i.e. straight venation segments between two junctions), and ℓ_j are their lengths. We chose this weighting to prevent over-represenation of very short segments that are sometimes spurious.

2.6 Topological distance

In order to quantify more precisely the difference between distinct leaf network topologies, we use the distributions of $\{q_j\}, \{L_e\}$ obtained for each network. A convenient similarity measure is the Kolmogorov-Smirnov statistic $D_{\mathrm{KS}} = \max_x |F(x) - G(x)|$, where x ranges over all possible values of q (L), and $F_{q,\ ,(L)}(x)$ $(G_{q,\,(L)}(x))$ are empirical cumulative probability distributions formed from the sets of $\{q_j\}$, $(\{L_e\})$ for a pair of leaf networks. KS distances were calculated using `scipy`'s `stats` package.

3 Principal Component Analysis

Let centered data set be $\mathbf{Y} = \mathbf{y} - \mu$, where $\mathbf{y}$ is the matrix of data points and μ the matrix of means. We scale our data such that all means are 0 and all standard deviations are 1, so as to remove scale

[1] `http://www.cvxopt.org`

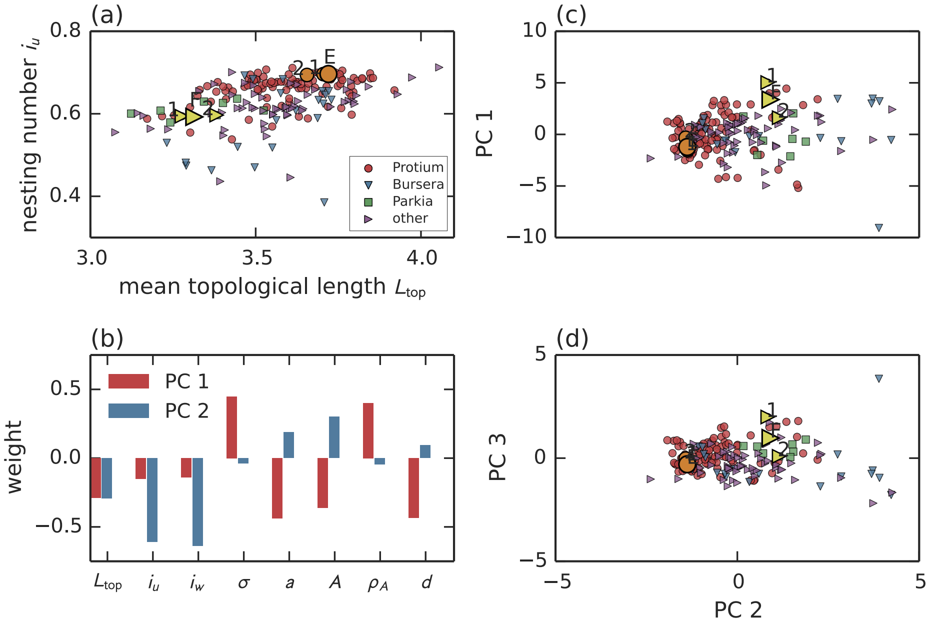

Figure 2: Principal Component Analysis of the leaf dataset. (a) Plot of the complete dataset in the space of mean topological length L_{top} and unweighted nesting number i_u. Marked are one leaf of *Protium grandifolium* and *Dalbergia miscolobium* together with their nearest neighbors (labeled as 1, 2) with respect to the statistical distance D_{KS} calculated from the local nestedness statistics $\{q_j\}$. (b) Weights of the eight geometrical and topological metrics in the PCA of the whole data set. Component 1 contains mostly the geometric metrics whereas component 2 contains mostly the topological metrics. (c, d) Plot of the whole dataset in the space spanned by the principal components 1 and 2 (in (c)) and 2 and 3 (in (d)). The marked leaves are the same as in panel (a).

	L_{top}	i_u	i_w	σ	a	A	ρ_A	d	rel. expl. var.
PC 1	−0.29	−0.15	−0.14	0.45	−0.44	−0.36	0.40	−0.43	55.9%
PC 2	−0.29	−0.61	−0.64	−0.04	0.19	0.30	−0.04	0.09	26.2%
PC 3	−0.54	0.36	0.16	0.20	0.23	0.42	0.47	0.25	9.3%
PC 4	−0.71	0.10	0.07	−0.31	−0.14	−0.39	−0.44	0.15	5.5%

Table 1: First four eigenvectors of the covariance matrix as well as the relative explained variance. The result from PCA is similar to Factor Analysis, showing that the most important principal components discriminate between geometric and topological metrics.

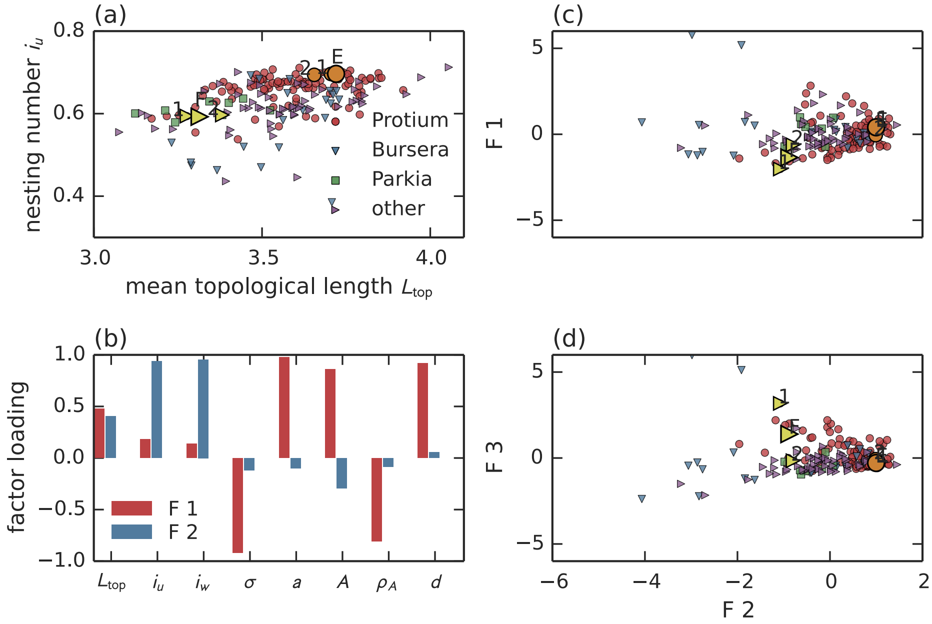

Figure 3: Factor Analysis of the leaf dataset. (a) Plot of the complete dataset in the space of mean topological length L_{top} and unweighted nesting number i_u. Marked are one leaf of *Protium grandifolium* and *Dalbergia miscolobium* together with their nearest neighbors (labeled as 1, 2) with respect to the statistical distance D_{KS} calculated from the local nestedness statistics $\{q_j\}$ (See also Figure 2). (b) Factor loadings of the eight geometrical and topological metrics in the FA of the whole data set. Factor 1 contains mostly the geometric metrics whereas factor 2 contains mostly the topological metrics. (c, d) Plot of the whole dataset in the space spanned by the factors 1 and 2 (in (c)) and 2 and 3 (in (d)). The marked leaves are the same as in panel (a).

effects from comparing data with different units. By calculating the eigen-decomposition of $\text{Cov}(\mathbf{Y})$ (equivalently the SVD of $\mathbf{Y}$), one obtains a new coordinate system in the data space whose axes are ordered such that (a) they are orthogonal, (b) they point in the directions with maximum variance. This results in a dimensional reduction when restricting to only the first few eigenvectors which explain the bulk of the variance in the data set. We performed PCA using `scikit-learn`'s `PCA` class [4]. We found that he first principal component contains mainly geometric leaf network features, whereas the second PC contains mainly topological features, thus confirming the interpretation that leaf network phenotypic space consists of the orthogonal directions, geometry and topology. For details see Table 1 and Figure 2.

4 Factor Analysis

Factor Analysis attempts to fit a linear latent variable model to the data. A factor model is given by

$$\mathbf{Y} = \Gamma \mathbf{z} + \epsilon. \tag{3}$$

	L_{top}	i_u	i_w	σ	a	A	ρ_A	d	rel. expl. var.
F 1	0.48	0.18	0.14	−0.92	0.97	0.86	−0.81	0.92	54.0%
F 2	0.40	0.94	0.96	−0.12	−0.10	−0.29	−0.09	0.06	25.9%
F 3	−0.20	0.20	0.08	0.32	0.15	0.37	0.54	0.05	8.1%
F 4	−0.27	0.09	−0.13	−0.06	−0.00	−0.06	0.08	0.21	2.0%
rel. noise var.	0.06	0.005	0.006	0.004	0.002	0.004	0.05	0.01	

Table 2: Factor loading matrix entries for the leaf data set as well as estimated noise variances. Factor Analysis was performed for four factors. Factor loadings vary between −1 and 1. The relative amount of total variance explained by the factors is given in the rightmost column. The amount of relative variance missing is explained by the relative noise variance.

Here, Γ is a matrix of factor loadings, $\mathbf{z}$ contains the latent variables (factors) describing the data set which are assumed to follow a Gaussian distribution with zero mean and unit variance, and ϵ is Gaussian noise. A simple calculation assuming uncorrelated factors with unit variance shows that the covariance matrix in the factor model is given by

$$\text{Cov}(\mathbf{Y}) = \Gamma^T \Gamma + \text{Cov}(\epsilon). \tag{4}$$

Fitting loadings Γ and noise ϵ is a hard optimization problem which can be tackled using expectation maximization, finding a maximum likelihood solution. This analysis was performed using the `scikit-learn 0.15.2` software [4] which implements algorithm 21.1 from [5] in its `FactorAnalysis` class. Figure 3 shows scatter plots in the space of the first three factors of a four-factor analysis as well as the factor loadings of the first three factors. Table 2 shows the complete loading matrix Γ, as well as the estimated noise variances for each variable. Using more than 4 factors does not result in a significant increase in explained variance, and only the first two factors have interesting interpretations.[2] It can be seen that the first factor contains almost exclusively contributions from geometric leaf traits, whereas the second contains almost exclusively contributions from topological leaf traits, establishing topology as an independent "direction" in the phenotypic space of leaf venation.

5 Pearson correlation analysis

In addition to the PCA and FA performed in the previous sections, we also performed classical Pearson correlation analysis between topological and geometrical leaf traits. The results are shown in Figure 4. They support the conclusions drawn from PCA and FA. The topological nesting number i_u is only very weakly correlated with the geometrical metrics, therefore it has almost its own principal component (PC 2). The topological length on the other hand shows some correlations with geometry, therefore it shows up significantly in both PC 1 and PC 2.

6 Linear Discriminant Analysis

Linear Discriminant Analysis (LDA) is a tool from supervised learning which attempts to find a set of hyperplanes optimally separating sets of points in a high dimensional space whose class membership

[2]Using 2 or 3 factors, we still find a strong divide between geometric and topological metrics in the most important factors.

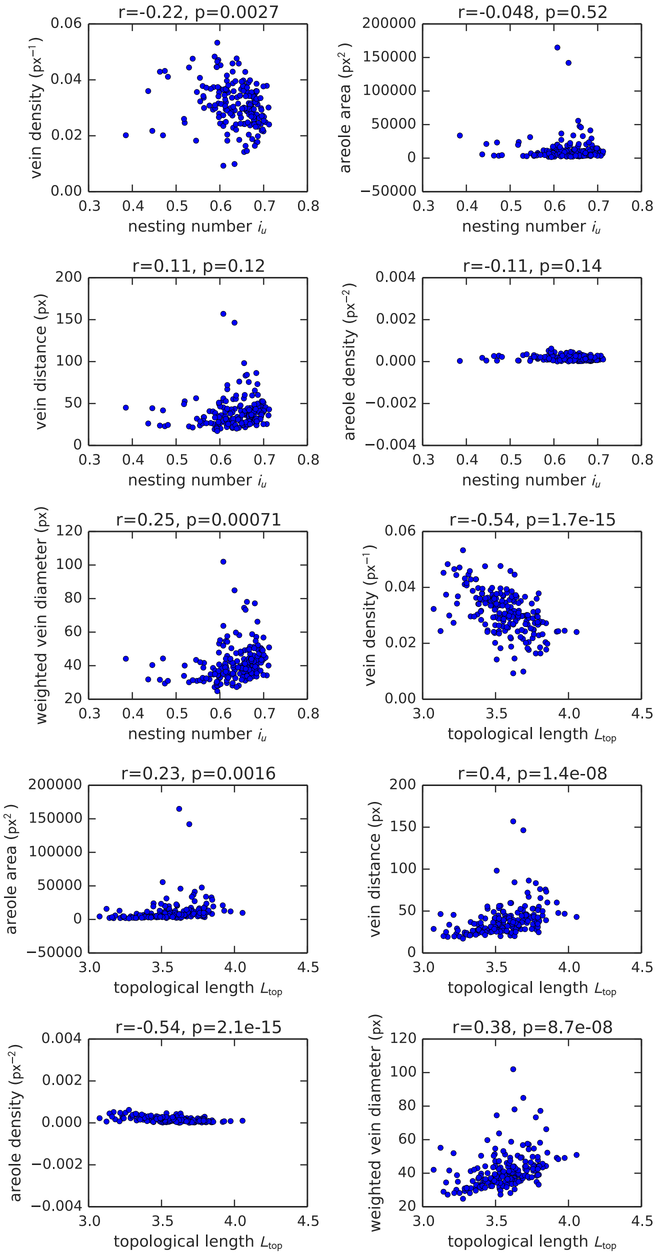

Figure 4: Pearson correlation analysis between topological and geometrical vein traits. We show the Pearson correlation coefficient r between pairs of vein traits as well as the associated p value. Nesting number i_u is only weakly correlated with geometric vein traits, Topological length L_{top} shows stronger correlations. This supports the conclusions drawn from PCA.

is known. LDA assumes that the probability distribution within each class is given by a Gaussian with mean μ_i and covariance matrix Σ_i, $P(\mathbf{x}|i) \sim \exp\left(-(\mathbf{x}-\mu_i)^T\Sigma_i(\mathbf{x}-\mu_i)/2\right)$, and that all class covariances are equal ($\Sigma_i = \Sigma$). This leads to linear equations defining the class boundaries. Class membership is defined by the decision function $G(\mathbf{x}) = \mathrm{argmax}_i\, P(\mathbf{x}|i)$. Relaxing the equal covariance assumption, one obtains Quadratic Discriminant Analysis (QDA), with quadratic manifolds as class dividers. Typically, QDA and LDA perform very similarly even when the equal covariance assumption is violated, so we restrict our analysis to LDA.

Typically, one evaluates the power of a classifier like LDA by dividing the data set into training and test sets. Then, the classifier is trained (its parameters are fit) using the training set, and a score such as accuracy *acc* (proportion of correctly classified data points) is obtained from the test set. This gives an estimate of the data set's separability (how well one can distinguish between the known classes based on the data).

We used `scikit-learn`'s implementation of LDA in the `LDA` class to assess the separability of different leaves based on geometric and topological metrics. In order to generate more data points, each leaf graph was cut into 3000×3000px fragments which were analyzed separately, calculating the same metrics as for the full leaves. For all 186 leaves, fragments were generated. Fragments with fewer than 128 areoles were discarded, leaving a total data set of 3446 fragments representing 183 leaves (some leaves were too small to be included in this analysis).

We then performed two tests, estimating performance using 10-fold stratified cross validation, calculating the accuracy score for the LDA estimator. Cross validation was done using the `cross_val_score` function in `scikit-learn`.

Full dataset test. In this test scenario, LDA was fit to the complete data set of leaf fragments. Because as a whole, leaf data forms a continuum without well-defined clusters, there is typically considerable overlap between data sets belonging to different leaves.

Using only geometrical degrees of freedom, we found an accuracy of 0.35 (95% CI: $[0.31, 0.39]$). Adding topology, we found a significant increase to 0.54 (95% CI: $[0.48, 0.60]$) (Welch's $t(15.6) = 15.8$, $p < 0.001$). So in this case, we found a performance increase of roughly 54% when using geometry and topology, vs. geometry alone, see Figure 5 (a) and Figure 6 (a).

We also test the effect of removing the smallest veins. For each of the leaf fragments, we remove the $p\%$ smallest diameter veins, calculate all topological and geometrical metrics and compute the same identification accuracy as for the fragments without removed veins. The results are plotted in Figure 7. Because the very smallest veins tend to be the freely ending veinlets, removing a relatively large portion does not change the topological metrics in a strong way. However, as soon as we remove more than $\approx 20--30\%$, topology becomes worse at improving identification accuracy. Accuracy due to geometric vein traits alone remains roughly constant.

Pairwise test. For each pair of individual leaves in the dataset, the same procedure was applied to obtain a mean pairwise accuracy score (the probability of correctly identifying a fragment as belonging to one of two leaves.) Because for some leaves there were fewer than 10 fragments, we chose a k-fold cross-validation scheme with $k = \min(n_a, n_b) - 1$, where $n_{a\,(b)}$ is the number of fragments in leaf $a\,(b)$. Again, using topological traits significantly improved the summary result (see Figure 5 (b) and Figure 6 (b)). The improvement per individual pair however was in many cases not large.

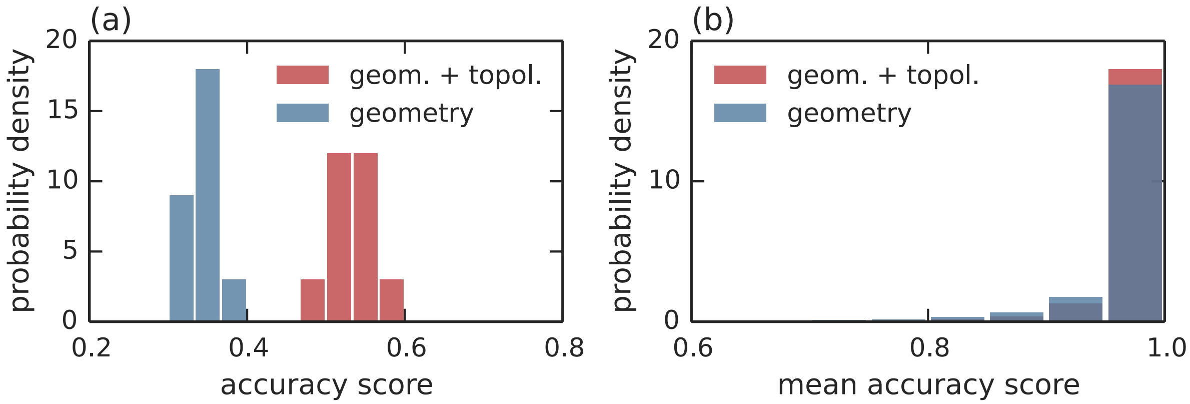

Figure 5: (a) Histograms of the distribution of Linear Discriminant Analysis accuracy scores obtained using 10-fold stratified cross-validation. Each leaf was fragmented into 3000×3000 pixel blocks, then all leaf traits were calculated for each block individually. LDA was used to fit hyperplanes optimally separating the data points using only geometry or geometry and topology. Since there is considerable variation already within a single leaf, accuracy of a simple classifier such as LDA is not expected to be high on a "difficult" problem such as this. Still, combining geometrical and topological information improves the classification accuracy by roughly 54%. (b) Histogram of Pairwise Linear Discriminant Analysis mean accucary score distribution. For each pair of leaves, LDA was used to fit an optimally separating hyperplane between the fragment data points, accuracy of the estimator was calculated using cross-validation. For individual pairs, accuracy largely improved, but not statistically significantly (Welch's t p value for most distributions > 0.05). Taking all pairwise accuracies together, the improvement is significant in distribution (KS $D = 0.07$, $p < 0.001$).

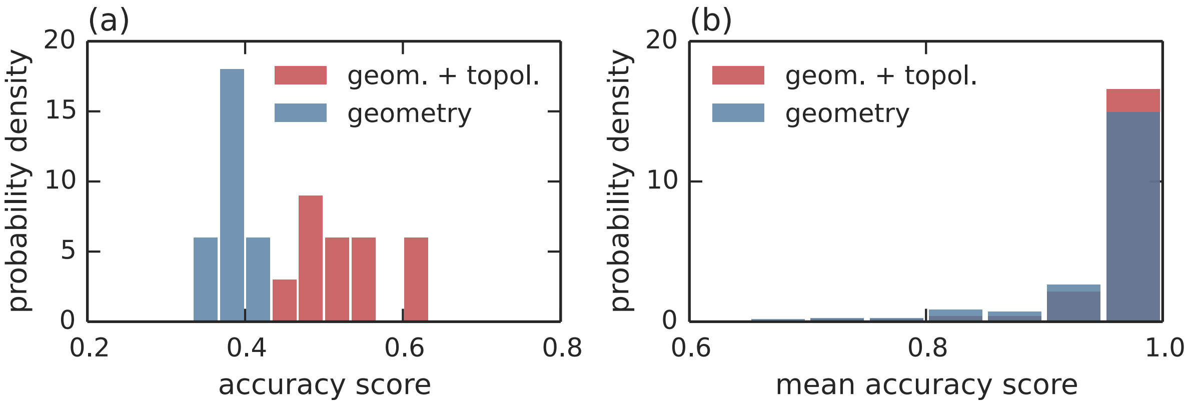

Figure 6: Analysis accuracy scores obtained using 10-fold stratified cross-validation for all species where more than one leaf specimen was available (80 specimen representing 33 species). Each leaf was fragmented into 3000×3000 pixel blocks, then all leaf traits were calculated for each block individually. LDA was used to calculate identification accuracies. (a) The accuracy that a leaf fragment is correctly identified as belonging to its species. Using geometry alone we find an accuracy of 0.38 (95% CI: $[0.33, 0.43]$), using geometry and topology the accuracy is 0.52 (95% CI: $[0.42, 0.63]$), a significant increase (Welch's $t(12.7) = 7.4$, $p < 0.001$). (b) For each pair of species the mean probability that one fragment is correctly identified to its species. The improvement is significant in distribution (KS $D = 0.21$, $p < 0.001$).

manual vein density (mm^{-1})	5.61	4.72	4.09	13.40	11.15	6.23	7.06	14.37	11.93	8.91
automatic vein density (mm^{-1})	5.94	4.96	4.56	11.81	8.38	5.14	8.21	10.65	11.01	7.99
relative error	0.06	0.05	0.11	−0.12	−0.25	−0.18	0.16	−0.26	−0.08	−0.10

Table 3: Comparison between manual and automatically estimated vein density.

7 Scaling of leaf traits with leaf area

Sack et al. [2] have identified many leaf traits which correlate with leaf area, even allowing to predict the original size of a leaf from analyzing a fragment. Here, as a form of validation for our vectorization methods, we perform a similar analysis for our dataset, reproducing the results of Sack *et al.* for geometric leaf traits (Figure 8). Similar to [2], we find that the main and major vein traits scale with leaf size whereas minor vein traits do not. This can be explained by noting that main veins form first during development and grow along with the leaf, whereas minor veins form last when the leaf lamina has already reached its final size, so their geometry should not scale with leaf area.

However, unlike geometric trains, leaf topology traits, as defined in our work, do no appear to correlate significantly with leaf size, further cementing them as an "orthogonal" direction in leaf phenotypic space.

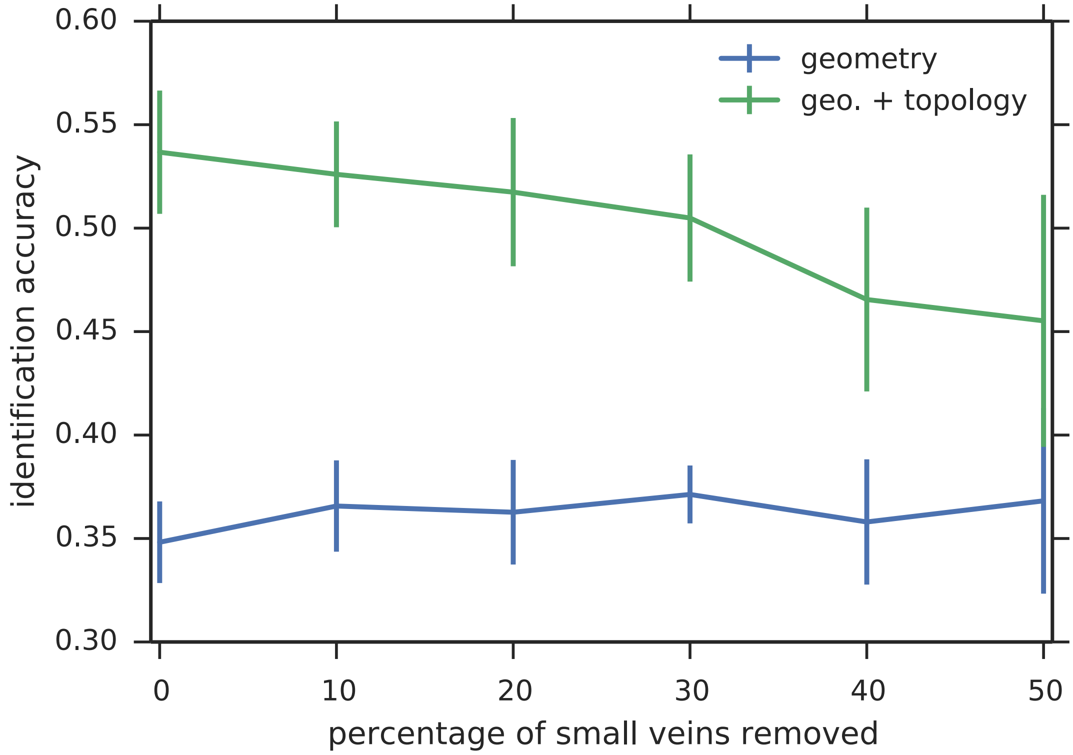

Figure 7: Change of identification accuracy when removing the $p\%$ smallest diameter veins from the leaf fragments. Accuracy from geometrical traits alone is roughly constant over the range between 0% and 50%, whereas accuracy from geometry and topology combined decreases. The error bars correspond to the standard deviation from 10-fold stratified cross-validation. Standard deviation from the combined vein traits increases with removing more small veins, corresponding to the fact that topology becomes less discriminatory.

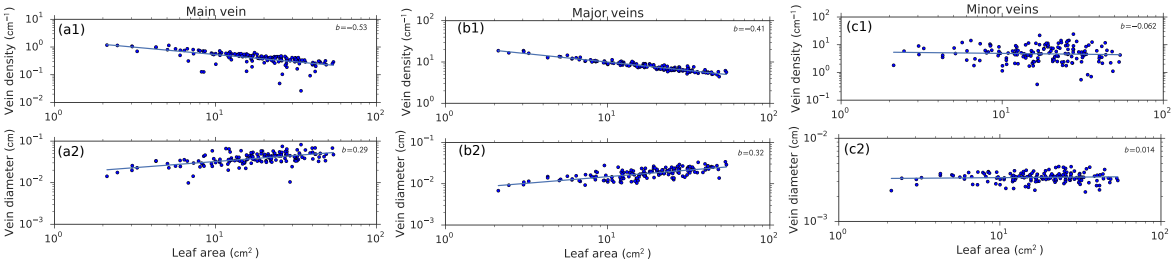

Figure 8: Scaling relations for vein characteristics. We plot the density and diameter of (a) main vein, (b) major veins (as defined in the text) and (c) minor veins with leaf area for all leaves in our dataset. In the main vein density plot, a few outliers can be spotted. These can be explained by the main vein detection algorithm not detecting the whole vein due to noise effects during vectorization. The red lines are linear least squares fits to the log data, the fitted slopes are (a1) main vein densities $b = -0.53$, 95% CI: $[-0.63, -0.43]$, $R^2 = 0.41$, $p < 10^{-18}$; (a2) main vein diameters $b = 0.29$, 95% CI: $[0.23, 0.36]$, $R^2 = 0.32$, $p < 10^{-13}$; (b1) major vein densities $b = -0.41$, 95% CI: $[-0.43, -0.39]$, $R^2 = 0.91$, $p < 10^{-80}$; (b2) major vein diameters $b = 0.32$, 95% CI: $[0.27, 0.37]$, $R^2 = 0.51$, $p < 10^{-24}$; (c1) minor vein densities $b = -0.062$, 95% CI: $[-0.22, 0.11]$, $R^2 = 0.003$, $p = 0.47$; (c2) minor vein diameters $b = 0.014$, 95% CI: $[-0.02, 0.05]$, $R^2 = 0.005$, $p = 0.41$.

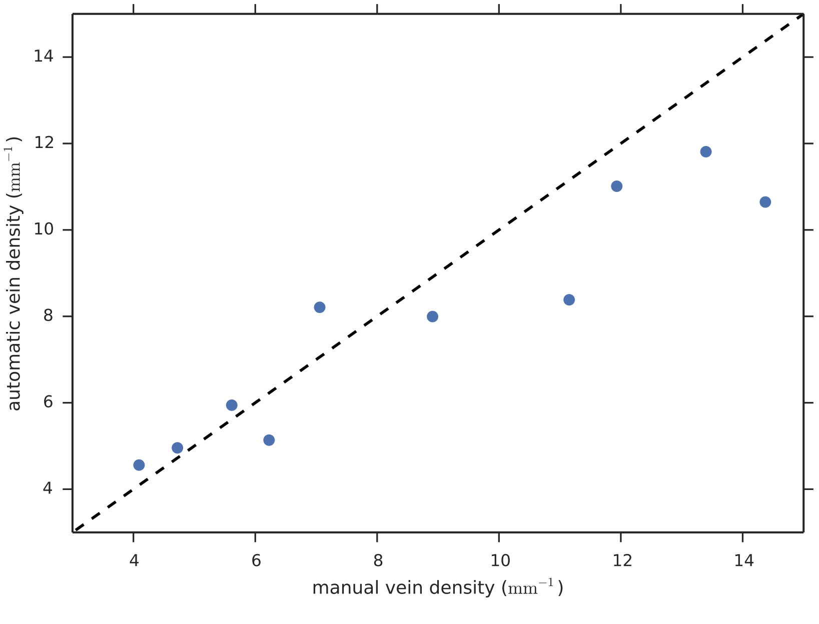

Figure 9: Comparison of manual vein density measurements with automatic measurements in 10 leaves of varying density. The dashed line is the curve $y = x$.

8 Vein density

In order to validate the method further, we manually measured vein density in small sections of the original scans off of the main vein using `ImageJ` by tracing veins using the *segmented line* tool. We compared these manual measurements with automatic measurements in similarly sized sections, one for each leaf, also away from the main vein. The results are shown in Figure 9 and Table 3.

For very dense leaves the automatic measurements tend to underestimate the manually derived density. This is due to the fact that very small veins cannot be discerned by the algorithm reliably due to lack of contrast or limited resolution in the original scans. We estimate that for leaves denser than $10\mathrm{mm}^{-1}$, vein density is not accurately calculated anymore. The mean absolute error is $-0.88\mathrm{mm}^{-1}$, the mean relative error is -0.06. Given that the topology calculations are robust to omission of the smallest veins, this limitation in our dataset does not affect significantly our results.

9 Leaf Vectorization

The extraction the networks from the original high-resolution scans (6400 dpi) can be divided into two main steps: segmentation of the image to create a suitable binary representation and skeletonization of the shapes. To segment the image we use a combination of Gaussian blurring to reduce noise, local histogram equalization and recombination with the original image to increase contrast, and Otsu thresholding [6] to find the optimal threshold for the creation of the binary image. For the skeletonization we use a vectorization technique known from optical sign recognition [7, 8]. The approach relies on the extraction and approximation of the foreground feature's contours using the Teh-Chin dominant point detection algorithm [9] and subsequent triangulation of the contours via constrained delaunay triangulation [10]. Therefore the foreground is partitioned into triangles which can be used to create a skeleton of the shape. Each triangle contributes a "center" point to the skeleton which is determined by looking for local maxima in the euclidean distance map [11] of the binary and together these centerpoints approximate the skeleton. By looking at edges shared between two triangles, neighborhood relations can be established and an adjacency matrix can be created. This adjacency matrix defines a graph composed of nodes (the former triangle centers) and edges (the connections between two adjacent triangles). In addition to the topology of the graph the original geometry of the network including coordinates of the nodes and lengths and radii of edges are preserved and stored in the graph. The processing is done using algorithms implemented in python. The framework uniting all the aforementioned functionality is called *NET* and can be found at `github.com/JanaLasser/network_extraction/`. Earlier digitization work [12] used other techniques and can be limited in terms of resolution [13, 14]. The process is visualized in Figure 10.

10 Hierarchical decomposition

The hierarchical decomposition algorithm itself is rather straightforward. However, given a weighted graph representing the venation network of a leaf (or even other planar networks), several preprocessing steps need to be taken before hierarchical decomposition *proper* can be applied. First, the graphs are visually inspected and regions containing obvious vectorization artifacts are removed. Then, the following automatic steps are applied.

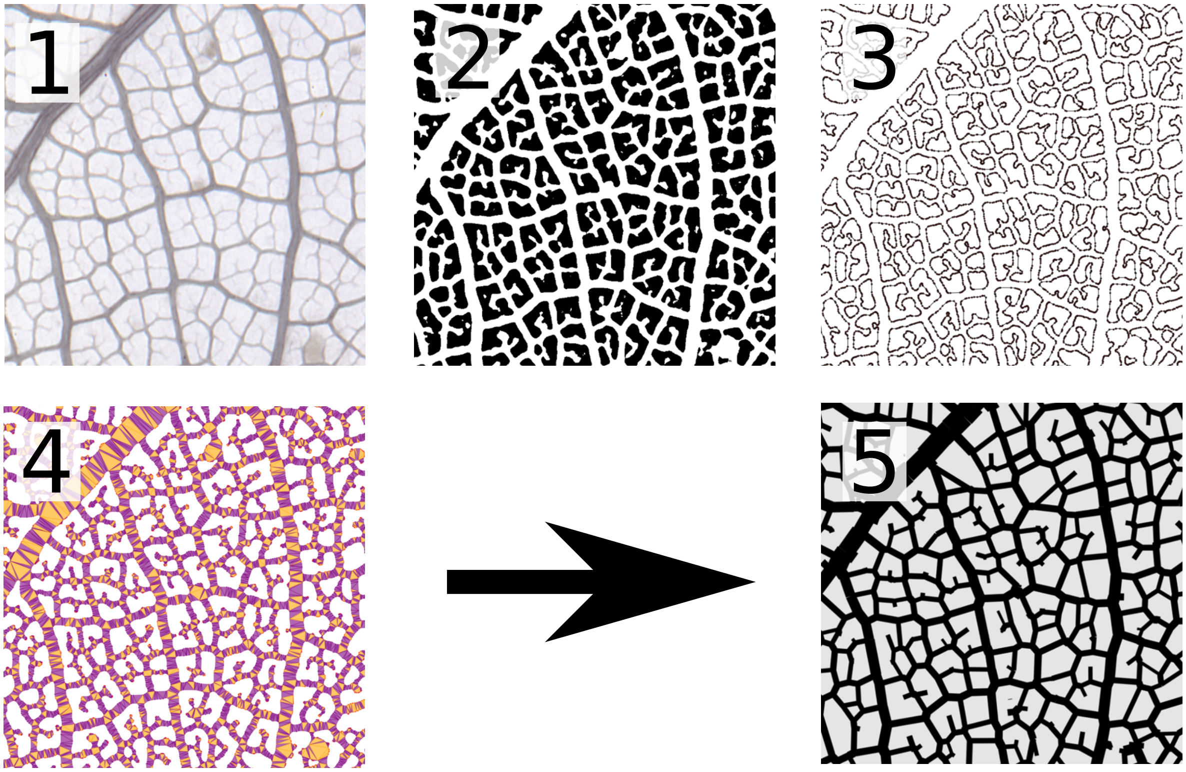

Figure 10: The vectorization process. (1) We start from a high resolution scanned image (6400 dpi) of the leaf. (2) A binary is generated using a combination of blurring, local histogram equalization and finally Otsu thresholding. (3) Teh-Chin dominant point detection is used to obtain a set of contour points. (4) Constrained Delaunay triangulation of the contour points. (5) The final graph representation of the vascular network. Although we are showing a small crop, the process was performed for a whole leaf.

10.1 Pre-processing

10.1.1 Removal of nonplanar artifacts

It is possible that during vectorization, crossings of some edges appear. This can be due to numerical errors or errors during creation of the graph skeleton. Luckily, such artifacts tend to be rare. They are removed by a heuristic method, iterating over all nodes n of the network and considering their 5-neighborhoods (i.e. the subgraph of all nodes which are at most 5 hops away from n). This is an arbitrary choice which works sufficiently well in practice[3]. For all pairs of edges in the 5-neighborhood, it is tested whether they intersect, and if they do, both edges are removed from the graph. Typically not more than one or two crossings (most of the time none) are detected in each whole leaf network.

10.1.2 Pruning of the graph

Hierarchical decomposition works only on the “loopy” part of a network. Therefore all subgraphs which cannot be expressed in terms of an (arbitrary) basis of cycles for the graph are removed. This cycle basis can be computed quickly with the help of `networkx`, but in general does not correspond to the basis of fundamental planar cycles needed for hierarchical decomposition. The parts of the network that can not be expressed a a combination of cycles in the cycle basis are discarded. This is equivalent to pruning any tree-like segments that are attached to the loopy backbone.

10.1.3 Choice of connected component

Hierarchical decomposition works on a per-connected-component basis. Therefore, we choose the largest connected component and discard the others for all subsequent steps. Small connected components are typically vectorization artifacts from imperfect binaries.

10.2 Hierarchical decomposition

In this section, we describe the actual hierarchical decompostion algorithm used in detail. Again, we can split up work into several sub-algorithms for the identification of the fundamental cycle basis, the construction of the dual graph and finally the extraction of the nesting tree.

10.2.1 Construction of fundamental cycle basis

The fundamental cycles hierarchical decomposition operates on are given by the facets of the graph G seen as a planar polygon composed of E edges and N vertices. A simple way of constructing such a basis is to traverse the graph in such a way that at each node n_i, the next node is chosen to be the left-most (or right-most) as seen from the vector $\vec{n}_{i-1,i}$, going from node $i-1$ to node i. This way, one can never leave the cycle belonging to one facet. This is shown in Figure 11. Traversal is complete once the same node is encountered that one had started with. This traversal needs to be done for all starting nodes and all starting directions to catch all facets of the graph. Since many will be traversed several times, equivalent facets are identified if they possess the same unordered set of nodes. The algorithm is stopped once $F = E - N + 1$ facets have been found, this being the total number of independent cycles in a graph with one connected component.

[3]To be guaranteed to work always, one would have to search the whole graph instead of a small neighborhood. This would be significantly slower.

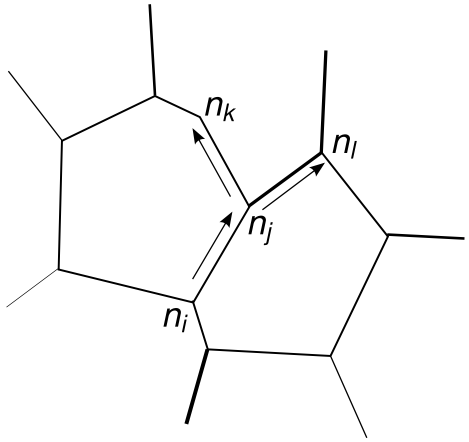

Figure 11: Detecting fundamental cycles (facets) by consistently taking the left-most path. (Left) The current node is n_j, and a decision needs to be made whether to proceed to n_k or n_l. To do this, first the unit vectors $\vec{n}_{ij}$ (pointing from point n_i to point n_j), $\vec{n}_{jk}$, and $\vec{n}_{jl}$ are formed. Then, we calculate $S_{k,l} = \mathrm{sign}(\sin\alpha_{k,l})(1-\cos\alpha_{k,l})$, where $\alpha_{k,l}$ is the angle between n_{ij} and n_{jk}, n_{jl}, chosen on the interval $[-\pi, \pi]$. The maximum value of $S_{k,l}$ is attained for the left-most direction as seen from n_j, coming from n_i. The corresponding node (n_k in this example) will be visited next.

10.2.2 Construction of cycle dual graph

A cycle dual graph G^* (or equivalently, the cycle adjacency matrix) is constructed to represent neighborhood relationships between the fundamental cycles. An efficient algorithm to achieve this proceeds by iterating through all cycles, storing for each edge in the cycle which cycle(s) it belongs to. This way, one can obtain for each edge a pair of cycles that the edge belongs to [4]. This information be used to construct a graph, or adjacency matrix, representing which cycles share an edge. Finally, the outer boundary cycle is removed, and each cycle sharing edges with the boundary is assigned its own virtual boundary cycle (replacing the single boundary cycle). This way, more information about the cycles on the network boundary is retained.

10.2.3 Hierarchical decomposition

The hierarchical decomposition algorithm proceeds as follows. We start with the cycle dual graph G^* and a set of disconnected, single nodes $S = \{c_i\}$ representing the fundamental facets that will eventually be leaf nodes of the nesting tree T. At each step,

1. we find a pair of cycles c_i, c_j from S such that $\min_{e \in c_i \cap c_j} R_e$ is minimal over all pairs of adjacent cycles.

2. we construct the cycle $c_k = c_i \triangle c_j$, where $\triangle$ denotes set-theoretic symmetric difference on the set of edges.

[4]Each edge can belong to at most two cycles in a planar graph.

3. we add a new node c_k to the tree T and connect it to the original cycles using the new edges $(c_i, c_k), (c_j, c_k)$.

4. we replace c_i, c_j by c_k in the cycle dual graph.

5. we repeat from step 1 until no more cycles remain in G^*.

The algorithm is visualized in Figure 1 of the main paper.

It is possible that cases appear in which taking the symmetric difference between two cycles results not in one new cycle, but two disconnected cycles. The result of this algorithm is a binary tree graph T, called the hierarchical decomposition tree, or nesting tree, representing the nesting structure of loops within the original graph G. We note that the map $G \mapsto T$ is not 1 to 1. Many inequivalent planar graphs map to the same nesting tree.

11 Developmental Model

Here we describe a phenomenological probabilistic model which simulates the formation of nested loops by means of growing rectangles which can divide. The model starts with a single rectangle. Each edge grows according to the equation

$$\frac{d\vec{x}_i(t)}{dt} = a\,\vec{x}_i(t), \tag{5}$$

which is exponential growth. All edges grow linearly in width, according to

$$\frac{dr_k}{dt} = b. \tag{6}$$

Here, a is the overall growth rate, b is the linear thickness growth velocity, r_k is the edge width of edge k and $\vec{x}_i$ is the node coordinates of node i.

In our model, there is a finite probability p_{split} for each rectangle to split in two at a given position and in time interval $[t, t+dt]$. This probability depends on the area A and a given target area A_0 as follows:

$$\begin{aligned} p_{\text{split}}(A) &= \frac{1}{2}\left(1 + \text{Erf}\left(\frac{A - A_0}{\sqrt{2}\sigma_A}\right)\right) &(7)\\ &= f\left(\frac{A - A_0}{\sigma_A}\right). &(8)\end{aligned}$$

Here, σ_A is a parameter which controls how likely it is for a rectangle to split before or after its area has reached A_0.

When a rectangle is divided, the position of the new edge is chosen such that it splits the rectangle along the long side. The position relative to one corner is

$$x_{\text{rel}} = \frac{1}{2} + \rho\,\text{rnd}\,. \tag{9}$$

Here, ρ is another parameter which controls how random the splitting position is where rnd is a uniform random number between $-1/2$ and $1/2$.

This model is based on four dimensional parameters, a, b, A_0, σ_A which can be combined using Buckingham's Π theorem to yield three dimensionless control parameters. These are $\alpha = \frac{b}{a\sqrt{A_0}}, \beta = \frac{\sigma_A}{A_0}$, ρ. This set of dimensionless control parameters shows that it is sufficient to vary A_0, σ, keeping a, b fixed. We choose the length and time scales $x_c = \sqrt{A_0}$, $t_c = a^{-1}$, obtaining the nondimensional set of equations

$$\frac{d\vec{x}_i(t)}{dt} = \vec{x}_i(t) \tag{10}$$

$$\frac{dr_k}{dt} = \alpha \tag{11}$$

$$p_{\text{split}}(A) = f\left(\frac{A-1}{\beta}\right). \tag{12}$$

These equations can easily be discretized and simulated. We choose to employ a simple forward Euler scheme with $\Delta t = 0.01$.

After the termination criterion has been reached (the final number of loops or total surface area has been reached), we add Gaussian noise with zero mean to all edge widths (vein thicknesses). The standard deviation is chosen to $f_n \, \mu_r$, where μ_r is mean edge width and f_n is a parameter. Edge widths may become negative during this procedure and such edges are removed from the network. The addition of this noise is meant to represent the measurement noise or intrinsic noise in the vein thicknesses. Simulations of growth conditions that produce nested vein structures but whose widths typically vary less than $f_n \, \mu_r$ will appear to have a more random, less highly nested topology.

Instead of splitting each rectangle into two, we also consider splitting it into three smaller rectangles at the same time. To model this, we include a probability $p_{\text{trif}} = 1 - p_{\text{bif}}$ for trifurcating each rectangle when it splits instead of simply bifurcating. The results do not change appreciably.

References

[1] Alejandra Vasco, Marcela Thadeo, Margaret Conover, and Douglas C. Daly. Preparation of Samples for Leaf Architecture Studies, A Method for Mounting Cleared Leaves. *Applications in Plant Sciences*, 2(9):1400038, 2014.

[2] Lawren Sack, Christine Scoffoni, Athena D McKown, Kristen Frole, Michael Rawls, J Christopher Havran, Huy Tran, and Thusuong Tran. Developmentally based scaling of leaf venation architecture explains global ecological patterns. *Nature communications*, 3(May):837, January 2012.

[3] Robert S Hill. A numerical taxonomic approach to the study of angiosperm leaves. *Bot. Gaz.*, 141(2):213–229, 1980.

[4] F. Pedregosa, G. Varoquaux, A. Gramfort, V. Michel, B. Thirion, O. Grisel, M. Blondel, P. Prettenhofer, R. Weiss, V. Dubourg, J. Vanderplas, A. Passos, D. Cournapeau, M. Brucher, M. Perrot, and E. Duchesnay. Scikit-learn: Machine learning in Python. *Journal of Machine Learning Research*, 12:2825–2830, 2011.

[5] D. Barber. *Bayesian Reasoning and Machine Learning.* Cambridge University Press, 2012.

[6] Nobuyuki Otsu. A threshold selection method from gray-level histograms. *IEEE Transactions on Systems, Man, and Cybernetics, Vol. SMC-9, No. 1*, 1979.

[7] K. C. Fan, D. F. Chen, and M. G. Wen. A new vectorization-based approach to the skeletonization of binary images. *Proceedings of 3rd International Conference on Document Analysis and Recognition*, page 627, 1995.

[8] Ju Jia Zou, Hung-Hsin Chang, and Hong Yan. A new skeletonization algorithm based on constrained delaunay triangulation. 2:927–930 vol.2, 1999.

[9] C. H. Teh and R. T. Chin. On the detection of dominant points on digital curve. *PAMI*, 11:859–872, 1989.

[10] L. P. Chew. Constrained delaunay triangulations. *Algorithmica*, 4:97–108, 1989.

[11] P. E. Danielsson. Euclidean distance mapping. *Computer Graphics and Image Processing*, 14:227–248, 1980.

[12] Charles A Price, Olga Symonova, Yuriy Mileyko, Troy Hilley, and Joshua S Weitz. Leaf extraction and analysis framework graphical user interface: segmenting and analyzing the structure of leaf veins and areoles. *Plant physiology*, 155(January):236–245, 2011.

[13] C. A. Price, P. R. T. Munro, and J. S. Weitz. Estimates of Leaf Vein Density Are Scale Dependent. *Plant Physiology*, 164(January):173–180, 2013.

[14] L. Sack, M. Caringella, C. Scoffoni, C. Mason, M. Rawls, L. Markesteijn, and L. Poorter. Leaf vein length per unit area is not intrinsically dependent on image magnification: avoiding measurement artifacts for accuracy and precision. *Plant Physiology*, 166(October):pp.114.237503–, 2014.
